# Law, Medicine, and Humanity

**Published:** 1893-10-21

**Authors:** 


### The Hospital

An Institutional Journal of

Science, Medicine, Nursing, and Philanthropy.

*For Hospitals, Asylums, Infirmaries, Dispensaries, Medical Practitioners, Students, and Nurses.*

Vol. XV.—No. 369.] [Oct. 21, 1893.

### LAW, MEDICINE, AND HUMANITY.

Law and medicine are contending over the living body of a political prisoner, Dr. Cornelius Herz, who is incarcerated in a Bournemouth lodging house, and it is not improbable that between them his life may be sacrificed. But law can have no desire to compass his death, because, even if he were proved guilty of all the charges made against him, not one of those charges is such as to demand the death penalty; and certainly medicine can have no wish to terminate his existence, for her business is to cure disease and to prolong life, without any regard whatsoever to merit or demerit in the character of the person.

This is a case which primarily concerns Dr. Cornelius Herz, but any living man, in Great Britain, France, or the world, who is of sufficient standing to be of a little political importance, may at any moment be placed in the position of Dr. Herz; and though he may be as innocent as Dr. Herz declares himself to be, or even as the babe unborn, his life may be sacrificed to a legal technicality. Clearly this is not in accordance with the dictates of reason and science. What agreement it may have with the broad and generous sentiments included within the four corners of the word "humanity" is still less apparent.

Scientific medicine, as represented by Dr. Lauder Brunton, Dr. William Frazer, and Mr. Malcolm McHardy, protests against this irrational anachronism; and so serious does it consider the situation to be that it adopts the very unusual procedure of uttering its protest in the *Times*. It appeals, in fact, from the helplessness of handicapped medicine and the pedantry of inelastic law to the sound and practical instincts of common humanity. The case, as we have said, primarily affects Dr. Cornelius Herz, and the facts may be stated in a very few words. The mere statement of them will reveal a condition of the British legal system which it behoves every justice-loving man to endeavour to put an end to. Briefly, then, Dr. Herz has been arrested in England, and charged with offences of a political character against the French law. But Dr. Herz, whose presence at Bournemouth was in the first instance, and still is, solely due to the state of his health, was not at the time of his arrest in a fit state to appear in a law court, and has never since been well enough. On the contrary, it is certified by men of such standing as that of Dr. Lauder Brunton that for Dr. Herz to appear in a law court would be to endanger his life. Nevertheless, the doctor has been willing and desirous that his case should be heard and argued in his absence. This, which seems so entirely reasonable a course, is the very procedure which, according to our law, cannot be acceded to. To the non-legal mind it seems the height of absurdity. That an accused person should object to be tried without himself being present in court one can readily understand. He might know a hundred things which would tell in his favour, and which might be known to no other living person than himself. But that the law should refuse to hear a man's case in the man's absence, even though he himself expresses his readiness for it to be so heard, and to abide by the result, seems to us nothing short of an unqualified absurdity. When in addition to this the compulsory attendance of the accused person, who in the present instance is almost certainly an absolutely innocent person, would result in the jeopardising of his life, it must be confessed that the "resources of civilisation," in so far as the British law is concerned, are of the most meagre and beggarly description.

The question is interesting to the medical profession from a medico-legal point of view. It is, in fact, a medical question first, and a legal question afterwards. But it is a public and a political question more than it is either medical or legal. What the public ought to see is this—that in cases of real and dangerous illness there ought to be interposed between the sick person and the stern arm of the law a medical barrier, a medical protection which should not only preserve the accused from the danger of personally appearing at a public trial, but which should deliver him from the almost equally dangerous wrong of an unrepelled accusation if he desires his case to be heard and decided in his absence. The public, we say, has in the medical profession a means of protecting itself, not indeed against the law, but against the harassing injustice of which the law may be made the willing or the unwilling instrument.

Of course, there are, and always have been, objections against conferring upon the medical profession as a whole such peculiar powers as may seem to be implied in the foregoing argument. But we do not propose that such powers shall be conferred upon the medical profession as a whole. Before indicating the nature of the proposal we have to make we will present one or two little regarded considerations which bear upon the question at the present time. Judges and lawyers have often expressed distrust of the medical profession because, as they say, "there are many doctors who will swear anything." This is, unfortunately, true, and it is a tradition of very long standing. But we submit two things for the consideration of the reasonable legal mind: First, that the doctors of the present day have a scientific competence which their predecessors did not possess; and, secondly, that not only is the character of the profession as a whole elevated above that of its predecessors in a remarkable degree, but that there is ample choice among hundreds of men of the highest character to enable a selection to be made of a medical tribunal which shall be absolutely above suspicion.

The case of Dr. Herz is in many respects a typical case. That is to say, there are hundreds, perhaps thousands, of persons every year whose lives are jeopardised, either as principals or as witnesses, by compulsory attendance in law courts. As the law stands at present these persons have no protection of an adequate kind. If one doctor certifies that a particular principal or witness is unable to appear in court, another doctor can be found who will as promptly certify, not only that he may appear without danger, but that a cross-examination by a leader of the Bar will be the best possible thing for his health. All this is unworthy of civilisation, much more of scientific reason. Our suggestion is that juries of competent doctors should be empanelled at convenient centres throughout the United Kingdom. Any principal or witness in a litigation case should have the right of appeal to such a jury, and its decisions should be absolutely final. If as in the case of Dr. Herz, there should be no early probability of the restoration to health of a principal or a witness, the case, if he so desired, should proceed in his absence, and it should not be assumed, as it almost invariably is now, that his absence is tantamount to a confession of the weakness or wickedness of his cause.

What we desire to insist upon, not only to doctors, but to lawyers and the general public, is that the scientific medicine of to-day is not like the medicine of old. It is a new thing, a new instrument of human guidance, human protection, and human well-being. It is an instrument of actual knowledge, actual experience, and actual truth. It is capable of making the law of the lawyer more just, of enlarging, without licence, the just liberty of the subject, and of protecting the sick and suffering from dangers of a peculiarly harrassing kind, the dangers that is to say of an inelastic law administered by minds of limited comprehensiveness. Surely the enlargement of freedom is a great object! But a far greater object to a justice-loving people is the improvement and amplification of the law in such wise that it shall gradually become in fact what it now is in theory, "the perfection of reason," and shall prove itself not only a swift and unerring sword for the punishment of the wrong-doer, but a father and unfailing protector of all that are helpless and oppressed.

